# Prognosis and diagnosis prediction of lung adenocarcinoma outcome based on a novel model anchored in circadian clock‐related genes

**DOI:** 10.1002/ccs3.12053

**Published:** 2024-11-13

**Authors:** Bernard Perbal

**Affiliations:** ^1^ International CCN Society Nice France

**Keywords:** circadian rhythm, clock‐related genes, lung adenocarcinoma, prognosis

## Abstract

The transition of JCCS from Springer to Wiley was rich in future prospects and was confirmed by the Journal reorganization launched in 2019. However, in spite of my recently renewed demand, the “B&B” section of the Journal (Bits and Bytes not be confused with Bed and Breakfast, albeit…) did not fit into the Wiley Publishing categories and had to be dropped, to the great disappointment of many authors and our readership. This Editorial will fill the gap by discussing new published aspects on the topic of the connections existing between circadian cellular signaling and cancer prognosis, with the identification of eight genetically significant clock‐related genes in lung cancer.

## Graphical abstract

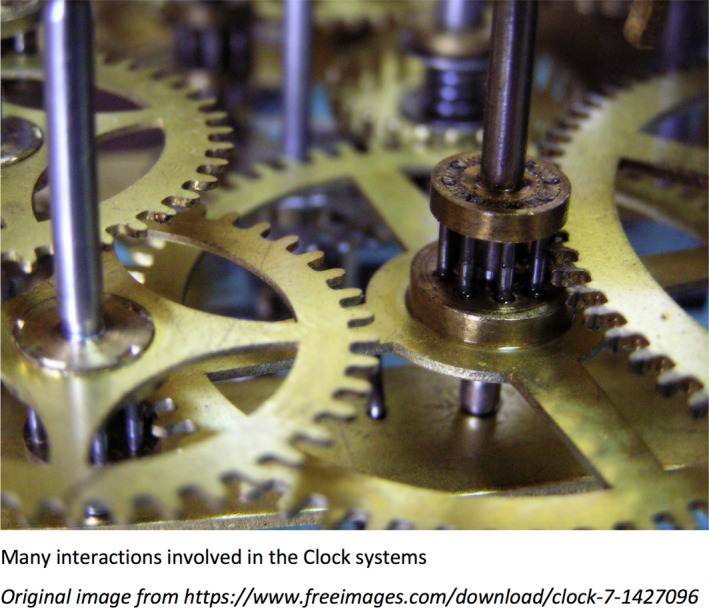

In a recent breakthrough manuscript, Sun et al.^1^ reported the construction of a risk model based on circadian clock‐related genes for lung adenocarcinoma prognosis, paving the road for the optimization of treatment decision‐making.

In normal conditions, the circadian system allows living organisms to adjust their behavior to daily time‐dependent environmental variations in an anticipated fashion.

Salient features of the circadian rhythm.

Briefly, natural oscillation of several biological functions that occur regularly in most species are under the control of intrinsic circadian clocks^2^ that were set as a result of an evolving adaptation to the 24‐h light–dark cycle. The circadian rhythms that are genetically encoded involve a molecular transcriptional feedback loop acting as an oscillator system. Manifestation of the intrinsic nature of the oscillator is provided by the experimental observation of Jean‐Jacques Ortous de Mairan, regarding the properties of *Mimosa pudica*. The French astronomer observed (presented in 1729 before the French Royal Academy by Marchant) that while *Mimosa pudica* “is sensitive to the sun or the day and their pedicles fold up at sunset, similarly as when we touch, or we shake the plant,” “this phenomenon does not require that the plant be outdoors or exposed to the sun.” The opening of the plant still persists when the plant is put into a sealed light‐proof container. The work of Ortous de Mairan, presented by J.J. Marchant (Figure 1), drew an interesting parallel with sick people who can feel the day or night from their bed without actually seeing the light.^3^ With this simple comment, Ortous de Mairan and Marchant insinuated that humans may have an endogenous clock as well. Their conclusions were reinforced by the observations of De Candolle.^4^ The study of abnormal behavior of *Drosophila* mutants exhibiting 24‐h alterations of both the cycle of pupal eclosion rhythm and the adult activity rhythm,^5^ led to the molecular identification of the period gene (per) subjected to circadian rhythm.^6^, ^7^, ^8^, ^9^ The PER protein self regulates its rhythmic expression^10^ and the first discovered mammalian clock gene was discovered in mice showing abnormal circadian behavioral patterns.^11^, ^12^ The PER protein was found to shuttle between the cell nucleus and cytoplasm in a temporally regulated manner.^13^ The cyclic degradation of PER is blocked by the binding of TIM (timeless) protein. The 2017 Nobel Prize in Physiology and Medicine was awarded jointly to Jeffrey C. Hall, Michael Rosbash, and Michael W. Young for their discoveries of molecular mechanisms that control circadian rhythms.

**FIGURE 1:**
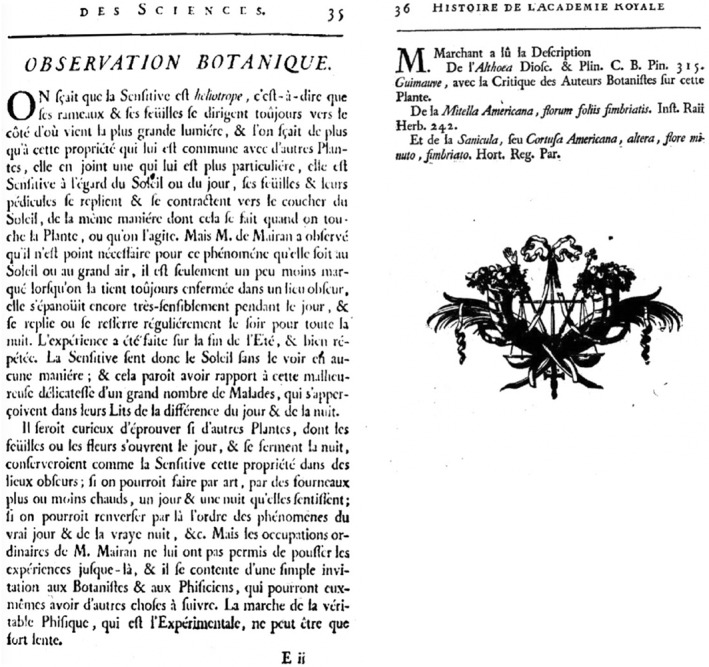
Original transcript of the Observation Botanique presented and published in 1729 at the Académie Royale des Sciences.

The molecular clockwork is based on clock genes participating in transcriptional/translational feedback loops (TTFLS). Two proteins known as CLOCK (CLK, for circadian locomotor output cycles protein kaput) and BMAL1 (Brain and Muscle Arnt‐like protein‐1) are in the center of mammalian TTFLS.

At the beginning of the day, CLOCK/BMAL1 heterodimers bind to the nuclear E box promoters of target genes that are activated. These targets include genes encoding the transcriptional repressors Period (Per1, 2, and 3) and Cryptochrome (Cry1 and 2). The resulting Period and Cry proteins first accumulate in the cytoplasm to form heterocomplexes that interact with casein kinase I (CKl delta and epsilon) and 5′AMP‐activated kinase (AMPK) and are degraded by the proteasome after phosphorylation. This provides the temporal delay required before inactivating the CLOCK/BMAL1 heterodimers.

In the evening, the PER/CRY complexes enter the nucleus to trigger an inactivation of the CLOCK/BMAL1 heterodimers, resulting in the transcriptional inhibition of PER and CRY genes. The TTFLS is closed (for details about accessory loops driven by the CLOCK/BMAL1 complex, see,^14^, ^15^, ^16^, ^17^). It is now established that the rhythmic oscillations are also driven by posttranscriptional and posttranslational regulatory mechanisms.

Pathological dysregulations of the 24‐h circadian rhythm have been associated with altered metabolism and cell cycle functioning, leading to cancer development and progression. Perturbations in circadian rhythms have been associated to heightened cancer risks and early mortality of patients suffering from cancers of breast, ovaries, liver, colon, pancreas, prostate, and lungs.^1^ Further details regarding the mechanisms controlling the circadian rhythm can be found in the advanced information scientific background published by Carlos Ibanez.^18^

Circadian rhythm disruption and health, lung adenocarcinoma prognosis.

The circadian rhythm is recognized as a multi‐oscillator system, governed by a central pacemaker in the hypothalamic suprachiasmatic nucleus and peripheral oscillators present in every organ of the body. Circadian misalignment may be the cause of pathological conditions and diseases, a situation exacerbated by occupational requirements including nightshift work or reduced light perception.

The circadian systems have been connected to the signaling of metabolic health^19^, ^20^ and to the expression of extracellular matrix (ECM) proteins.^21^ A crosstalk between the molecular clock and transcriptional regulation of matrix genes was found to govern both, ex vivo and in vivo, the expression of genes encoding the CCN proteins. The transcription of CCN family genes was also affected by the circadian rhythms, becoming altered with age at both the behavioral and molecular levels.

It is known for several years that health consequences induced by circadian rhythm disruption result in higher risks of lung cancer.

Sun et al.^1^ undertook an exploration of the possible relationships existing between circadian clock‐related genes and the outcome of lung adenocarcinoma (LUAD), which is the most prevalent histological subtype of non‐small cell lung cancer (NSCLC), accounting for a large share of morbidity and mortality. It originates predominantly from the bronchial mucosal epithelium and less frequently from the mucous glands of the larger bronchi.

At the present time, and according to the authors, surgery is currently the most effective treatment for early‐stage LUAD. There is an acute need to establish an effective model to supersede and replace the traditional approach that is hampered by the lack of specificity of these tumors frequently diagnosed at a late stage.^22^

Based on the evidence in support of circadian rhythms disruption playing a key role in tumorigenesis by facilitating the establishment of cancer hallmarks.^23^ The authors first used the DESeq2 algorithm to search for genes differentially expressed in LUAD samples. It provides a more comprehensive view of RNA‐seq (RNA sequencing) data than traditional microarray technology. Briefly, DESq2 uses a statistical tool widely utilized in bioinformatics to measure the expression levels of all genes in different samples. Because it takes into account and corrects for the inherent variability in the sequencing data, it provides more accurate results.

The statistical model calculates the difference in gene expression between two or more groups of samples, and fits a negative binomial distribution to each gene, after estimating the variance of gene expression levels. Then DESeq2 calculates the *p*‐value for each gene when applied to identify differentially expressed genes, with a smaller *p*‐value indicating stronger evidence for differential expression. The typical workflow offered by DESq2 involves statistical analysis, data visualization, pathway analysis, and interpretation.

A total of 5382 differentially expressed genes (DEGs) were identified between the gene expression data (expression and clinical) of 535 LUAD and 59 normal lung samples from The Cancer Genome Atlas Lung Adenocarcinoma Collection (TCGA) at (https://portal.gdc.cancer.gov/).

Venn diagrams (allowing to calculate intersections between lists of elements) performed with the group of 5382 DEGs and 276 circadian clock‐related genes were retrieved from the MsigDB databases (https://www.gsea‐msigdb.org/gsea/). The gene Set Enrichment Analysis permits establishing whether “*an a priori defined set of genes shows statistically significant, concordant differences between two biological states (e.g., phenotypes*).”

In the present case, 76 genes where found to be related to circadian rhythm.

Among these, 16 genes significantly correlated with overall survival were identified as potential prognostic markers. Further to LASSO regression and multivariate Cox regression statistical analysis, eight potential prognostic markers (ADRB1, BMAL2, LGR4, NPAS2, PTGDS, RORA, SFTPC, and TYMS) were identified. A prognostic model using these eight circadian clock‐related genes (CCRGs) was constructed. Among these genes, the expression levels of four genes (BMAL2, LGR4, NPAS2, and TYMS) were negatively correlated with OS (overall survival), whereas the expression levels of four genes (ADRB1, PTGDS, RORA, and SFTPC) were positively correlated with OS.

According to the authors, their pioneering approach facilitates the precise prognostic assessment of patients afflicted with LUAD. Their very comprehensive approach has “successfully unearthed eight differentially expressed genes that are closely linked to the circadian rhythm in the context of LUAD and have opened up innovative avenues for tailoring personalized treatment strategies, and in turn, promise to elevate the overall standard of care and management for individuals grappling with lung adenocarcinoma.”

A set of complementary studies performed by Wang et al^24^ have established and validated “a signature for lung adenocarcinoma based on metabolism‐related genes.”

Altogether these new approaches should provide better prognostic tools in the near future that will hopefully permit development of a successful management approach for these tumors.

For those who like to read news while having breakfast in bed, this Editorial might sound like a challenging B&B…

## CONFLICT OF INTEREST STATEMENT

The author declares no conflicts of interest.
